# Breathing mode selectively modulates brain-wide functional connectivity

**DOI:** 10.1371/journal.pone.0334165

**Published:** 2025-11-14

**Authors:** Sadeq Mohammadi, Gholam-Ali Hossein-Zadeh, Mohammad Reza Raoufy

**Affiliations:** 1 Department of Bioelectric, School of Electrical and Computer Engineering, College of Engineering, University of Tehran, Tehran, Iran; 2 School of Cognitive Sciences, Institute for Research in Fundamental Sciences (IPM), Tehran, Iran; 3 Department of Physiology, Faculty of Medical Sciences, Tarbiat Modares University, Tehran, Iran; 4 Institute for Brain Sciences and Cognition, Tarbiat Modares University, Tehran, Iran; Museo Storico della Fisica e Centro Studi e Ricerche Enrico Fermi, ITALY

## Abstract

While respiration is known to rhythmically modulate brain activity, how different breathing modes (nasal vs. oral) affect frequency-specific large-scale neural connectivity in humans remains unexplored. We used resting-state functional magnetic resonance imaging (fMRI) to examine how nasal and oral breathing modulate functional brain connectivity, focusing on blood oxygenation level-dependent (BOLD) fluctuations in the intermediate frequency band of 0.1–0.2 Hz in 20 healthy male participants. A fully data-driven ROI-based inference approach across 133 whole-brain ROIs revealed that nasal and oral breathing significantly activated the olfactory region and brainstem, respectively. Seed-based connectivity (SBC) analysis, using nonparametric permutation testing (10,000 iterations) and cluster-wise false discovery rate (FDR) thresholding (p-FDR < 0.05), based on these seeds, revealed distinct patterns of network engagement depending on breathing mode. Nasal breathing was associated with greater functional connectivity within higher-order brain networks, including the salience, somatosensory, default mode, and frontoparietal networks. Conversely, oral breathing increased connectivity centered on the brainstem, engaging subcortical regions involved in autonomic regulation and survival functions. Despite these differences, both conditions recruited stable respiratory core regions comprising the hippocampus, amygdala, and insula. These findings suggest a novel framework, the respiration-entrained brain oscillation network (REBON), defined by three operational criteria: (1) it is frequency-specific to the 0.1–0.2 Hz band (centered around ~0.16 Hz); (2) the activity of its principal regions, the olfactory region and brainstem, alternates in dominance depending on the mode of breathing; and (3) it includes a stable core of limbic and interoceptive structures, such as the hippocampus, amygdala, and insula. Understanding this network may have implications for future therapeutic strategies aimed at supporting cognitive functions, emotion regulation, and the integrity of large-scale brain networks in both clinical and wellness contexts; however, these translational implications require validation in future experimental studies.

## 1. Introduction

With each breath through the nose, airflow modulates oscillatory activity in the brain, whereas oral breathing bypasses this direct sensory input and its associated neural influence [1–3]. Nearly eight decades ago, Edgar Adrian first documented that rhythmic oscillations in the olfactory bulb of hedgehogs were synchronized with the breathing cycle, laying the groundwork for understanding the fundamental coupling between respiration and brain function [4]. Building on this, a study in 2003 demonstrated that slow oscillatory activity in the olfactory cortex of rats was closely tied to respiratory rhythms [5]. Grosmaitre et al. (2007) further improved this understanding by showing that olfactory sensory neurons (OSNs) serve a dual function, not only detecting odors but also acting as mechanical sensors that respond to airflow [6]. This dual functionality highlights how nasal airflow activates neural pathways that extend beyond olfactory processing.

Building on these foundational works, Zelano et al. (2016) used intracranial EEG to demonstrate that nasal respiration modulates oscillatory activity in limbic structures, particularly the amygdala and hippocampus [3]. Since then, a growing body of evidence has shown that nasal respiration entrains widespread neural oscillations in both olfactory and non-olfactory regions [7–13]. Furthermore, recent studies employing rhythmic nasal air-puff stimulation have demonstrated modulation of activity in multiple brain regions, including the medial prefrontal cortex (mPFC) and hippocampus, leading to improvements in working memory [14]. In addition to these findings, direct stimulation of the olfactory pathway in rodent models has been shown to enhance gamma-band coherence and mitigate Alzheimer’s-like pathology by strengthening functional connectivity within memory-related networks [15]. Most recently, electrical stimulation of the olfactory epithelium was found to restore disrupted OB–mPFC–hippocampal connectivity, normalize excitatory/inhibitory balance in the hippocampus, and rescue memory performance in mechanically ventilated rats [16]. This finding is crucial as it demonstrates that, rather than being driven by respiration per se, these effects may result from targeted stimulation of the olfactory epithelium, activating the olfactory pathway like the mechanical stimulation provided by nasal airflow during natural breathing. This suggests that direct activation of the olfactory pathway could contribute to neural regulation and the preservation of cognitive function, even in the absence of natural breathing.

In contrast, oral breathing bypasses the OSNs and eliminates mechanical stimulation of the olfactory bulb, thereby disrupting a key pathway for sensory respiration entrainment [3,6]. Without nasal input, central respiratory pattern generators in the brainstem, such as the preBötzinger complex, remain active and continue to generate intrinsic respiratory rhythms [17,18]. These brainstem structures are widely considered to function as central pacemakers, producing slow oscillations that propagate via ascending neuromodulatory pathways and modulate cortical excitability, arousal regulation, and motor activity [12,19]. Unlike the olfactory pathway, which provides bottom-up sensory feedback entrained by airflow, the brainstem route may engage more internally regulated dynamics associated with homeostatic control and autonomic regulation [1,17]. Previous studies have shown that this pathway plays a critical role in maintaining physiological stability and coordinating brain-body communication [12,20].

Although increasing evidence emphasizes the influence of breathing mode on brain function [21], to the best of our knowledge no human studies have explicitly tested the hypothesis, using the intermediate frequency (IMF) band (0.1–0.2 Hz) of resting-state fMRI, that mechanically activated OSNs during nasal breathing contribute differently to brain-wide functional connectivity than internally driven brainstem oscillations during oral breathing. Critically, detecting respiration-entrained brain oscillations requires the selection of an appropriate frequency band. The ~ 0.16 Hz subrange has been proposed as a key coordination frequency that synchronizes respiratory, cardiovascular, and neural systems. This subrange is considered a preferred frequency within the binary hierarchy model of neurophysiological rhythms [19,22]. Accordingly, the 0.1–0.2 Hz range, with ~0.16 Hz at its center, is of particular interest. This frequency band is thought to support neural synchrony and may reflect the influence of a central oscillator that modulates large-scale brain connectivity [23].

To address this gap, we introduce the concept of the Respiration-Entrained Brain Oscillation Network (REBON): a large-scale, frequency-specific (0.1–0.2 Hz) network of brain regions characterized by a shift in its central node, from the olfactory region during nasal breathing to the brainstem during oral breathing. While the overall spatial topology of REBON is flexible, it includes a stable core of regions that remain consistently engaged across breathing modes (such as key limbic and interoceptive structures), alongside a flexible periphery composed of higher-order networks whose connectivity is selectively modulated by the active central node. This network is hypothesized to reflect neural entrainment to rhythmic respiration and is distinct from canonical resting-state networks due to its sensitivity to breathing modality and its mechanosensory origin via olfactory input [21,24].

This study employed resting-state fMRI and a fully data-driven analytical approach to systematically compare the nasal and oral breathing conditions within the specific frequency band (0.1–0.2 Hz). By isolating the effects of breathing route on whole-brain functional connectivity, we aimed to reveal how distinct nasal and oral breathing pathways selectively organize large-scale network topology. This approach provides a novel framework for understanding how the mode of breathing sculpts brain activity and may have implications for cognitive regulation, emotional balance, and clinical interventions involving breath modulation.

## 2. Methods

### 2.1. Participants

Twenty-five healthy, right-handed adult male participants (age: 23.9 ± 2.18 years) with no self-reported physical or mental health disorders were enrolled for this study. Prior to the experiment, each participant’s health status was verified using the General Health Questionnaire-28 (GHQ-28) [25,26] and the Depression Anxiety Stress Scales-42 (DASS-42) [27,28], with all participants scoring within the normal range on these assessments. Exclusion criteria were: (1) history of neurological or somatic disorders, including neurodegenerative disease; (2) traumatic brain injury; (3) respiratory diseases or nasal cavity disorders; (4) history of COVID-19 infection; (5) history of smoking or drug/alcohol abuse; (6) cardiovascular disorders; (7) claustrophobia; (8) metal implants; (9) any structural abnormalities on T1-weighted images (inspected by a neuroradiologist expert); (10) head motion exceeding 2 mm of displacement or 2 deg rotation in any direction.

The study protocol was approved by the Ethics Committee of Tarbiat Modares University [IR.MODARES.REC.1400.175]. The recruitment period spanned from 15 May 2022–4 January 2023, during which all participants were screened and enrolled in accordance with the approved study protocol. All participants reviewed and signed a written informed consent form and were not informed of the study’s aims to maintain blinding. All methods were performed in accordance with relevant guidelines and regulations, including the Declaration of Helsinki.

### 2.2. Experimental design

Data were collected from each subject in a single session, with all sessions conducted at 1 p.m. on Saturdays through Wednesdays. Initially, a high-resolution structural image was acquired. Subsequently, each subject underwent three resting-state fMRI runs, each lasting 10 minutes. During one run, subjects were instructed to breathe normally and only through their nose, while in another run, they were instructed to breathe normally only through their mouth. The third run involved nasal air-puffs during oral breathing and was not included in this study. Adherence to nasal breathing was ensured using a mouth tape, and oral breathing was confirmed with a soft silicone nose clip. To minimize carryover effects and ensure physiological recovery, a 10–15 minute interval was maintained between the runs, and the order of runs was randomized across subjects to minimize potential order effects. Prior to each 10-minute resting-state fMRI run, participants spent five minutes breathing in the same mode (nasal or oral) assigned to the upcoming run to stabilize their breathing patterns.

During the scans, subjects were instructed to keep their eyes open and fixate on a white crosshair presented on a dark screen. Foam pads were used to stabilize the head and reduce motion artifacts, while noise-canceling headphones were provided to enhance comfort and mitigate scanner noise. For correction of EPI distortions, field map scans were acquired prior to each resting-state fMRI run. Electrocardiogram (ECG) and respiration signals were monitored using Physiological Monitoring Unit (PMU Wireless Physio Control, Siemens, Erlangen, Germany), to ensure a natural heart rate (HR) and breathing pattern.

### 2.3. Data acquisition

Structural and functional MRI data acquisition were performed using a 3-Tesla Siemens Prisma scanner (Siemens, Erlangen, Germany) located in the National Brain Mapping Lab (NBML), Tehran, Iran, using a 20-channel head coil. Structural images were acquired using a T1-weighted (T1w) magnetization-prepared rapid acquisition gradient echo (MPRAGE) sequence with the following parameters: voxel size: 1 × 1 × 1 mm; matrix size: 256 × 256 × 176; TR: 2000 ms; TE: 3.47 ms; flip angle: 7 deg. Resting-state fMRI data were acquired using a T2*-weighted gradient-echo echoplanar imaging (single-shot EPI) sequence with the following parameters: voxel size: 3 × 3 × 4 mm; matrix size: 70 × 70 × 34; TR: 2000 ms; TE: 30 ms; flip angle: 90 deg. Additionally, field map acquisitions were performed using a dual-echo gradient-recalled echo (GRE) sequence with the following parameters: voxel size: 3.75 × 3.75 × 3 mm; matrix size: 64 × 64 × 38; TR: 476 ms; TE: 4.92/7.38 ms; flip angle: 60 deg. Simultaneously with the fMRI scans, ECG and respiration signals were recorded using PMU.

### 2.4. Quality assessment

Beyond the initial quality control performed during scanning, additional data quality assessments were conducted using the MRIQC tool [29]. Prior to using MRIQC, the MRI data were formatted in compliance with the Brain Imaging Data Structure (BIDS) standard [30]. MRIQC generated detailed visual reports for both structural and functional MRI data, which were manually reviewed to identify artifacts such as artifactual structures in the background, susceptibility distortion artifacts, motion-induced signal dropouts, and aliasing ghosts [31]. While most data met quality standards, three subjects were excluded from further analysis: two due to exceeding head motion criteria, and one as a result of a structural artifact in the crown region, as indicated by motion peaks in the carpet plot [31,32].

### 2.5. Physiological signals analysis

Before preprocessing, the quality of raw respiration and ECG signals was visually inspected and verified. Respiration frequency (RF) and HR were then extracted for each subject using the PhysIO toolbox [33] in MATLAB (The MathWorks Inc., USA) and were manually checked and corrected. To ensure a homogeneous dataset, the difference in RF and HR between conditions was calculated for each subject, and outliers were identified using a threshold of two times the interquartile range (IQR). Two subjects were excluded based on RF outliers, resulting in a final sample of 20 subjects (age: 24 ± 2.31 years). A paired permutation test with 100,000 iterations was subsequently performed to assess whether the differences in RF and HR between the two conditions were statistically significant.

To further evaluate potential differences in heart rate variability (HRV) as a proxy for autonomic state between the two breathing conditions, we computed the root mean square of successive differences (RMSSD), a widely used time-domain metric of vagally mediated HRV [34]. RR intervals were derived from the ECG signal using the PhysIO toolbox, and the RMSSD was calculated for each subject and condition. Similar to the RF and HR analysis, we applied a paired permutation test to assess whether RMSSD differed significantly between nasal and oral breathing.

### 2.6. Preprocessing

The first five volumes were discarded to allow for magnetic field stabilization. Image preprocessing was performed using fMRIPrep 20.2.6 (RRID: SCR_016216) [35,36], which is based on Nipype 1.7.0 (RRID: SCR_002502) [37,38]. The T1w images were corrected for intensity non-uniformity (INU) with N4BiasFieldCorrection [39], distributed with ANTs 2.3.3 (RRID:SCR_004757) [40], and used as the T1w reference throughout the workflow. The T1w reference was then skull-stripped with a Nipype implementation of the antsBrainExtraction.sh workflow (from ANTs), using OASIS30ANTs as the target template. Brain tissue segmentation of cerebrospinal fluid (CSF), white matter (WM), and gray matter (GM) was performed on the brain-extracted T1w using fast (FSL 5.0.9, RRID:SCR_002823) [41]. Volume-based spatial normalization to standard spaces (MNI152NLin6Asym) was performed through nonlinear registration with antsRegistration (ANTs 2.3.3), using brain-extracted versions of both T1w-reference and the T1w template.

For each resting-state fMRI dataset, the following preprocessing was performed. First, a reference volume and its skull-stripped version were generated using a custom methodology of fMRIPrep. A fieldmap was estimated based on a phase-difference map calculated with a dual-echo GRE sequence, processed with a custom workflow of SDCFlows and further improvements in HCP Pipelines [42]. The fieldmap was then co-registered to the target EPI reference run and converted to a displacements field map (amenable to registration tools such as ANTs) with FSL’s fugue and other SDCflows tools. Based on the estimated susceptibility distortion, a corrected EPI reference was calculated for more accurate co-registration with the anatomical reference. The BOLD reference was then co-registered to the T1w reference using bbregister (FreeSurfer 6.0.1) [43], which implements boundary-based registration [44]. Co-registration was configured with six degrees of freedom. Head-motion parameters with respect to the BOLD reference (transformation matrices, six corresponding rotations, and translation parameters) are estimated using mcflirt (FSL 5.0.9) [45]. BOLD runs were slice-time corrected using 3dTshift from AFNI 20160207 (RRID:SCR_005927) [46]. The BOLD time series were resampled onto their original, native space by applying a single composite transform to correct for head motion and susceptibility distortions and were resampled into MNI standard spaces (MNI152NLin6Asym) with a 2 mm isotropic resolution. The quality of the preprocessed data was evaluated using fMRIPrep reports, and the unsmoothed fMRI data in MNI space were prepared.

Finally, the preprocessed fMRI data from fMRIPrep were imported into the CONN toolbox [47] (RRID:SCR_009550, release 22.v2407) [48], which requires SPM12 (RRID: SCR_007037, release 12.7771) [49] and MATLAB, and then smoothed using spatial convolution with an 8 mm full-width at half-maximum (FWHM) Gaussian kernel.

### 2.7. Denoising

In the next step, the smoothed functional data underwent a denoising pipeline [50] that included regression of potential confounding effects characterized by six motion parameters (three translations and three rotations) and linear trends (two factors) within each functional run, followed by band-pass frequency filtering of the BOLD time series [51] between 0.1 Hz and 0.2 Hz. Notably, no retrospective noise correction methods, such as RETROICOR [52] or CompCor [53] were applied. This decision was based on growing evidence that such techniques can suppress low-frequency BOLD fluctuations that reflect meaningful respiration-entrained neural activity, which were intentionally preserved in line with the primary aim of the study [13,54]. A recent review further highlights that physiological signals carry genuine respiration-related neural components, and their removal during denoising may inadvertently eliminate relevant neural oscillations associated with cognitive and functional brain processes [21].

### 2.8. Functional connectivity analysis

The primary objective of this study was to investigate how nasal breathing, via mechanical stimulation of OSNs, differentially modulates whole-brain connectivity patterns compared to oral breathing. To address this, and without making any prior assumptions about ROIs, a fully data-driven approach was adopted. First, a whole-brain ROI-based inference analysis was performed to identify significant regions and their connectivity patterns. Based on the identification of two significant ROIs, two seed-based connectivity (SBC) analyses were conducted, each targeting one of the detected ROIs. All analyses were performed using the CONN toolbox.

#### 2.8.1. ROI-based inference.

In the first-level analysis, ROI-to-ROI connectivity (RRC) matrices were estimated characterizing the functional connectivity between each pair of regions among 133 ROIs, comprising 91 cortical and 15 subcortical regions from the Harvard-Oxford atlas [55], 26 cerebellar regions from the AAL atlas [56], and one manually added olfactory ROI from the AAL atlas. Abbreviations and full names of all 133 ROIs are listed in S1 Table. Functional connectivity strength was computed as bivariate correlation coefficients between the denoised BOLD time series of each ROI pair and then Fisher Z-transformed. These correlations were derived from a general linear model (GLM) [57], estimated separately for each ROI pair. The full Fisher Z-transformed ROI-to-ROI connectivity matrices for the nasal and oral breathing conditions are provided in S1 Data and S2 Data, respectively.

Group-level analyses were performed using a GLM [58] employing the ”ROI-based inferences“ option in the CONN toolbox. In this approach, a separate multivariate model was estimated for each ROI by grouping together all connectivity values between that ROI and the remaining ROIs. Specifically, each row of the ROI-to-ROI connectivity matrix was treated as a cluster. This method enables statistical inference at the level of individual ROIs by evaluating whether the overall connectivity pattern from a given ROI differs significantly between conditions.

Since this study employed a within-subjects design, each subject contributed one connectivity value per condition (nasal and oral breathing), with breathing condition serving as the independent variable. For each ROI, an F-statistic was computed to test the null hypothesis that the overall pattern of connectivity did not significantly differ across conditions. The resulting ROI-level p-values were corrected for multiple comparisons using the FDR procedure [59]. ROIs were considered significant at a threshold of *p*-FDR < 0.05, and to further characterize their connectivity profiles of each significant ROI, a post-hoc connection-level threshold of uncorrected *p* < 0.01 was applied to identify individual connections showing the strongest condition-related effects.

#### 2.8.2. Seed-based connectivity.

Seed-based connectivity (SBC) maps were estimated to characterize the spatial pattern of functional connectivity with a seed region. Two independent SBC analyses were performed, each using one of the two seed regions identified from the ROI-based inference results (the brainstem and the olfactory region). At the first-level analysis, functional connectivity strength was computed as bivariate correlation coefficients between the denoised BOLD time series of the seed region and every voxel across the brain, followed by Fisher Z-transformation. These correlations were derived from a GLM [57], estimated separately for each seed-to-voxel pair.

Group-level analyses of SBC were performed using a GLM [58]. For each individual voxel, a separate GLM was estimated, with first-level connectivity measures at this voxel as dependent variables. Since this study employed a within-subjects design, each subject contributed one connectivity measurement per condition (nasal and oral breathing), and the breathing condition served as the independent variable. Voxel-wise hypotheses were evaluated using multivariate parametric statistics with random-effects modeling across subjects and covariance estimation across conditions. Inferences were performed at the level of individual clusters as groups of contiguous voxels. Cluster-level inferences were based on nonparametric statistics from randomization/permutation analyses [60,61], with 10,000 residual-randomization iterations. Results were thresholded using a combination of a cluster-forming *p* < 0.01 voxel-level threshold and a familywise corrected *p*-FDR < 0.05 cluster-mass threshold [62].

### 2.9. BOLD–physiological variability analysis

To evaluate whether potential differences in physiological variability between conditions could account for the observed connectivity effects, we conducted a control analysis assessing the relationship between BOLD fluctuations and cardiorespiratory variability. Specifically, we examined associations between BOLD signals from ROIs and two physiological parameters: respiratory volume per time (RVT) and heart rate variability (HRV).

For each subject, using the PhysIO toolbox, RVT was estimated from the respiratory signal by calculating the amplitude difference between an inhalation and the subsequent exhalation extrema (breath depth), divided by the time interval between them (breath duration), and HRV was derived from the cardiac beat-to-beat interval time course [33]. To model the influence of physiological fluctuations on the BOLD signal, the RVT and HRV time series were convolved with their respective response functions, the respiration response function (RRF) [63] and the cardiac response function (CRF) [64], as implemented in the toolbox, resulting in two physiological regressors: RVT and HRV.

We then computed Pearson correlation coefficients between each physiological regressor and the BOLD signals from each of the 133 ROIs, separately for the nasal and oral breathing conditions. For each subject and ROI, the correlation coefficients were Fisher Z-transformed to normalize their distributions. Paired-sample t-tests were then performed across subjects to compare the Z-transformed correlations between the nasal and oral breathing conditions at each ROI. Finally, resulting p-values were corrected for multiple comparisons using FDR, and significant effects were identified at *p*-FDR < 0.05. All analyses were implemented in MATLAB.

### 2.10. Respiration–BOLD phase-locking analysis

To empirically validate the relationship between respiration and BOLD oscillations within the 0.1–0.2 Hz frequency band, we performed a phase-locking analysis between the respiration signal and BOLD activity in the condition-specific regions identified through the ROI-based inference analysis: the olfactory region (during nasal breathing) and the brainstem (during oral breathing). Phase-locking was assessed using the phase-locking value (PLV), a normalized measure, ranging from 0 to 1, that quantifies the consistency of the phase lock between two signals over time, independent of the absolute phase difference. The PLV is computed by averaging vectors representing phase differences on the unit circle for all samples within a sliding window (Eq 1) [65]:


$${PLV}_{xy}=\frac{\text{1}}{N}\left|\sum_{n=\text{1}}^{N}e^{j\theta \left(x(n),\;\;y(n)\right)}\right|$$ (1)

where *x* and *y* are two time series signals, $\theta \left(x(n),\;y(n)\right)=(\varphi_{x(n)}-\varphi_{y(n)}\backslash )$ is the phase difference at sample *n*, and *N* is the number of samples in the sliding window. A PLV close to 1 indicates strong phase synchrony, whereas a value near 0 suggests random or inconsistent phase relationships.

Our phase-locking analysis was based on the PLV approach, following the strategy used by [66]. To prepare the respiration signal for analysis, it was first band-pass filtered between 0.01 and 0.25 Hz (anti-aliasing filter) and then downsampled to match the BOLD sampling rate. PLV was then estimated using cross-wavelet analysis, implemented via the “Cross Wavelet and Wavelet Coherence” toolbox [67] in MATLAB. Excluding the small unreliable segments due to the cone of influence (COI) and the constraints imposed by the window size at the beginning and end of the time series, PLV was calculated from instantaneous phase differences within the 0.1–0.2 Hz band using sliding windows corresponding to approximately four cycles at the center frequency of ~0.16 Hz. This window length has been recommended as “a suitable compromise between bias/noise and temporal resolution” [66].

Statistical significance was assessed using a surrogate data approach based on the Hilbert transform, following the S4 method described in [68]. Specifically, 1,000 surrogate phase-difference series were generated by estimating the instantaneous phase and frequency within the 0.1–0.2 Hz band, permuting the frequency vector, and reconstructing new phase signals. The S4 method preserves both the temporal structure (the power spectrum of the instantaneous frequency) and the phase-slip dynamics of the original signal, providing a rigorous and conservative framework for testing against false-positive phase-locking effects. The 95th percentile of the PLV distribution derived from these surrogate data was used as the significance threshold at a p-value of 0.05. To quantify the duration of significant phase-locking, we calculated the “%sigbins” metric, defined as the percentage of time samples showing statistically significant phase coupling (*p* < 0.05) for each subject and condition [66]. A phase-locking effect was considered reliable if %sigbins was greater than or equal to 10%, following the criteria proposed in [69].

To ensure that our PLV results were not driven by aliasing of faster respiratory rhythms into the 0.1–0.2 Hz band, we conducted a control analysis in which the respiratory signal was downsampled without applying an anti-aliasing filter, thereby allowing potential higher-frequency components to fold into the target band. Specifically, we first applied a broader band-pass filter (0.01–0.5 Hz) to the respiration signal and then downsampled it to match the BOLD sampling rate. PLV values between BOLD and aliased respiration signals were then recalculated to test whether the observed coupling could be explained by aliasing rather than by genuine respiration–BOLD interactions.

## 3. Results

### 3.1. Comparison of physiological metrics between conditions

We first assessed whether the nasal and oral breathing conditions differed in basic physiological parameters. The mean ± standard deviation of RF was 0.31 ± 0.04 Hz during nasal breathing and 0.30 ± 0.05 Hz during oral breathing. HR averaged 69.10 ± 11.26 bpm and 69.49 ± 10.00 bpm, respectively. RMSSD was 17.16 ± 6.74 ms for nasal breathing and 17.53 ± 4.92 ms for oral breathing. Paired permutation tests (100,000 iterations) revealed no significant differences in RF, HR, or RMSSD (p = 0.18, 0.59, and 0.7, respectively). All subject-level physiological metrics (RF, HR, and RMSSD) for each breathing condition are provided in S3 Data.

### 3.2. ROIs with significant connectivity

Using ROI-based inference analysis, two of the 133 ROIs showed significant differences in functional connectivity between the nasal and oral breathing conditions: the olfactory region and the brainstem. Connectivity was significantly stronger in the olfactory region, with all post-hoc thresholded connections occurring during the nasal breathing condition. In contrast, the brainstem showed significantly greater connectivity, with all post-hoc thresholded connections identified under the oral breathing condition. A statistical summary of these two significant ROIs is presented in Table 1.

**Table 1:** Statistical summary of the two significant ROIs identified by ROI-based inference.

Analysis Unit	Statistic (F(2,18))	p-uncorrected	p-FDR
Brainstem	15.21	0.000136	0.018054
Olfactory	12.35	0.000420	0.027930

**Fig 1:**
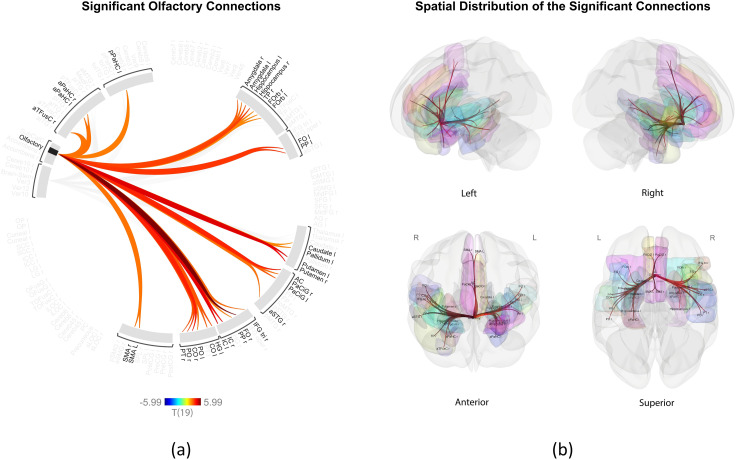
Enhanced olfactory connections during nasal breathing compared to oral breathing. **(a)** Connectome ring visualization showing statistically significant functional connections identified by ROI-based inference analysis (nasal > oral contrast) between the olfactory region and 34 cortical and subcortical ROIs. Warm-colored connections indicate stronger connectivity during nasal breathing (p < 0.01). Color intensity reflects the t-values, as indicated by the color bar (range: −5.99 to +5.99). Non-significant regions are shown in gray. **(b)** 3D glass brain representations illustrate the anatomical distribution of significantly connected ROIs from four views (left, right, anterior, and superior). ROIs are semi-transparently colored by anatomical region, and red lines indicate significant connections corresponding to those in the connectome ring. Full ROI names and abbreviations are provided in S1 Table. All plots were generated using the CONN toolbox.

Fig 1 illustrates the significant connectivity patterns of the olfactory region, including a connectome ring visualization (Fig 1a) and 3D glass brain representations from four views (Fig 1b). All 34 significant connections for this region showed increased connectivity during nasal breathing, encompassing widespread cortical and subcortical regions, such as included regions within the frontal lobe (frontal operculum and frontal orbital cortex), insular cortex, amygdala, hippocampus, parahippocampal cortex, and basal ganglia. A statistical summary of these connections is provided in Table 2.

**Table 2:** Statistical summary of the 34 ROIs functionally connected to the olfactory region in the nasal > oral breathing contrast.

#	Connected ROI	Statistic (t(19))	p-unc	p-FDR	#	Connected ROI	Statistic (t(19))	p-unc	p-FDR
**1**	FO r	5.99	0.000009	0.001201	**18**	IFG tri r	3.28	0.003964	0.027331
**2**	Putamen l	4.87	0.000105	0.006051	**19**	Amygdala l	3.25	0.004257	0.027331
**3**	IC l	4.76	0.000138	0.006051	**20**	PaCiG r	3.24	0.004273	0.027331
**4**	Putamen r	4.53	0.000228	0.007532	**21**	aSTG r	3.20	0.004680	0.027331
**5**	IC r	4.30	0.000390	0.010287	**22**	aPaHC l	3.19	0.004830	0.027331
**6**	CO r	4.12	0.000584	0.011753	**23**	PO l	3.16	0.005102	0.027331
**7**	AC	4.09	0.000623	0.011753	**24**	SMA L	3.16	0.005166	0.027331
**8**	FO l	3.89	0.000974	0.015828	**25**	SMA r	3.15	0.005284	0.027331
**9**	PP l	3.84	0.001114	0.015828	**26**	aTFusC r	3.13	0.005525	0.027331
**10**	PO r	3.80	0.001199	0.015828	**27**	aPaHC r	3.12	0.005591	0.027331
**11**	Hippocampus r	3.74	0.001398	0.016780	**28**	PP r	3.04	0.006675	0.031234
**12**	PT r	3.53	0.002220	0.022428	**29**	pPaHC l	3.03	0.006862	0.031234
**13**	HG l	3.52	0.002303	0.022428	**30**	Pallidum l	3.00	0.007294	0.032095
**14**	Hippocampus l	3.50	0.002379	0.022428	**31**	TP r	2.93	0.008626	0.035664
**15**	CO l	3.41	0.002941	0.023286	**32**	FOrb l	2.93	0.008646	0.035664
**16**	PaCiG l	3.40	0.002984	0.023286	**33**	FOrb r	2.89	0.009482	0.037354
**17**	Amygdala r	3.40	0.002999	0.023286	**34**	Caudate l	2.88	0.009621	0.037354

In contrast, the brainstem showed significantly increased connectivity with 20 ROIs during oral breathing, primarily involving regions such as the basal ganglia, thalamus, amygdala, hippocampus, insula, and parahippocampal cortex. Fig 2a illustrates these connectivity patterns using a connectome ring, and Fig 2b shows a 3D glass brain visualization from the four views. Table 3 presents a statistical summary of these 20 significant connections.

**Table 3:** Statistical summary of the 20 ROIs functionally connected to the brainstem in the nasal > oral breathing contrast.

#	Connected ROI	Statistic (T(19))	p-unc	p-FDR	#	Connected ROI	Statistic (T(19))	p-unc	p-FDR
**1**	Amygdala r	−4.78	0.000130	0.007390	**11**	pITG l	−3.37	0.003183	0.038194
**2**	aPaHC r	−4.73	0.000145	0.007390	**12**	Caudate r	−3.31	0.003678	0.038431
**3**	Hippocampus l	−4.67	0.000168	0.007390	**13**	pTFusC l	−3.30	0.003785	0.038431
**4**	Hippocampus r	−4.17	0.000517	0.016382	**14**	Putamen r	−3.14	0.005369	0.047810
**5**	aPaHC l	−4.09	0.000621	0.016382	**15**	Thalamus r	−3.11	0.005757	0.047810
**6**	pTFusC r	−3.77	0.001284	0.025757	**16**	Putamen l	−3.11	0.005795	0.047810
**7**	Amygdala l	−3.74	0.001400	0.025757	**17**	pITG r	−3.07	0.006338	0.049212
**8**	Caudate l	−3.69	0.001561	0.025757	**18**	TP l	−2.99	0.007508	0.055062
**9**	aTFusC r	−3.51	0.002365	0.032839	**19**	IC r	−2.96	0.008094	0.055362
**10**	PP l	−3.48	0.002488	0.032839	**20**	pPaHC r	−2.94	0.008388	0.055362

**Fig 2:**
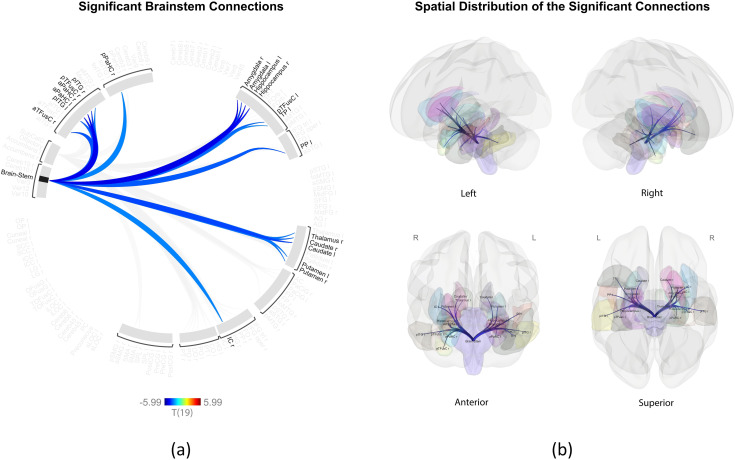
Enhanced brainstem connections during oral breathing compared to nasal breathing. **(a)** Connectome ring visualization showing statistically significant functional connections identified by ROI-based inference analysis (nasal > oral contrast) between the brainstem region and 20 cortical and subcortical ROIs. Cold-colored connections indicate stronger connectivity during oral breathing compared to nasal breathing (p < 0.01). Color intensity corresponds to the t-value, as indicated by the color bar (range: −5.99 to +5.99). Only significant connections are displayed; non-significant regions are shaded in gray. **(b)** 3D glass brain representations illustrate the anatomical distribution of significantly connected ROIs from four views (left, right, anterior, and superior views). ROIs are semi-transparently colored by anatomical region, and blue lines indicate significant connections corresponding to those in the connectome ring. A complete list of ROI abbreviations and full region names is provided in S1 Table. All plots were generated using the CONN toolbox.

### 3.3. Seed-based connectivity pattern

Two SBC analyses using the olfactory region and brainstem as seeds, identified large significant clusters with distinct connectivity patterns associated with the two breathing modes. Table 4 presents the cluster statistics identified in the SBC analyses for both seeds: one major cluster for the olfactory seed and two for the brainstem.

**Table 4:** Cluster statistics from the seed-based connectivity analysis for the olfactory and brainstem seeds.

Seed	Peak (x, y, z)	Cluster Size	Cluster p-FDR	Cluster Mass	Mass p-FDR
Olfactory	+34 + 6 −2	22748 voxels	0.000268	295463.19	0.000281
Brainstem	+28 −20 −24	4586 voxels	0.005284	62827.90	0.003915
	−18 + 4 −24	3409 voxels	0.005284	46282.21	0.004359

**Fig 3:**
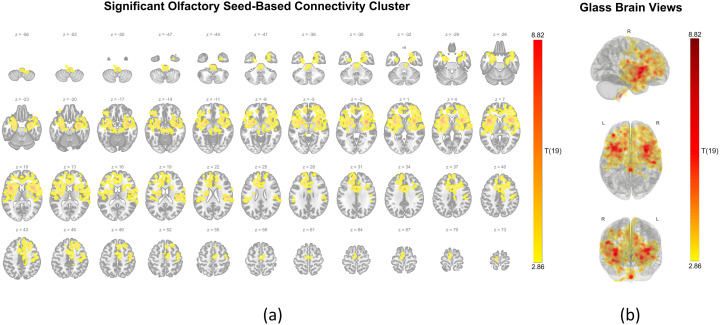
Olfactory seed connectivity: Cluster showing increased coupling during nasal breathing compared to oral breathing. **(a)** Axial slices showing a significant cluster where connectivity with the olfactory seed was greater during nasal breathing than oral breathing. Results were thresholded at the voxel level (p < 0.01) and corrected at the cluster level (p-FDR < 0.05). Warm-colored overlays represent voxels with positive t-values (t(19)), where higher values reflect stronger connectivity during nasal breathing. Z-coordinates are labeled in MNI space for anatomical reference. **(b)** Glass brain views present maximum intensity projections of the significant cluster from three perspectives: right lateral (top), superior (middle), and anterior (bottom). The color scale (range: 2.86 to 8.82) represents the magnitude of t-value, illustrating the spatial extent and bilateral distribution, primarily in frontal regions, of enhanced connectivity during nasal breathing. All plots were generated using the CONN toolbox.

The SBC analysis using the olfactory region as a seed revealed a single extensive cluster (22,748 voxels) centered at coordinates (+34, + 6, −2), with significantly greater connectivity during nasal breathing. This cluster involved widespread cortical and subcortical regions, including the frontal pole, frontal orbital cortex, frontal operculum cortex, central opercular cortex, precentral gyrus, supplementary motor area, cingulate gyrus, insular cortex, hippocampus, amygdala, putamen, and even brainstem. Fig 3a displays the spatial distribution of the significant olfactory SBC cluster on axial slices, while Fig 3b shows maximum intensity projection statistics from right lateral, superior, and anterior views, highlighting the spatial extent and widespread distribution in the frontotemporal regions. A summary of regions for which this cluster covers more than 10% of their volume is presented in Table 5, and the full composition is detailed in S2 Table.

**Table 5:** Summary of the significant cluster composition from the seed-based connectivity analysis of the olfactory region (covering ≥ 10% of the regions).

#	Region	Voxel Count	% Region Covered	#	Region	Voxel Count	% Region Covered
**1**	AC	1052	41	**23**	IFG tri r	221	40
**2**	IC l	905	68	**24**	Amygdala l	217	66
**3**	FP r	838	10	**25**	PT r	209	48
**4**	IC r	766	57	**26**	Hippocampus l	203	27
**5**	Brainstem	709	17	**27**	IFG oper r	195	28
**6**	Putamen l	650	75	**28**	FO l	190	54
**7**	CO r	552	63	**29**	PP l	190	53
**8**	CO l	550	56	**30**	Amygdala r	187	55
**9**	PaCiG l	541	41	**31**	aPaHC l	184	32
**10**	Putamen r	513	64	**32**	aPaHC r	175	27
**11**	TP r	499	21	**33**	PO l	162	29
**12**	PreCG r	429	10	**34**	HG l	152	49
**13**	PaCiG r	405	30	**35**	SMA L	148	23
**14**	FOrb l	393	23	**36**	PP r	139	37
**15**	FOrb r	337	23	**37**	Caudate l	129	24
**16**	PO r	337	63	**38**	IFG tri l	111	17
**17**	SFG r	330	12	**39**	aSTG r	83	30
**18**	FO r	281	90	**40**	aTFusC r	83	28
**19**	MidFG r	275	10	**41**	Pallidum l	75	25
**20**	TP l	272	11	**42**	IFG oper l	74	10
**21**	SMA r	236	33	**43**	HG r	62	22
**22**	Hippocampus r	228	33	**44**	aSTG l	39	14

**Fig 4:**
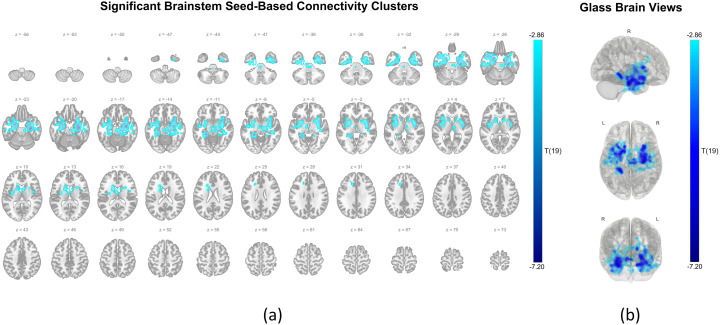
Brainstem seed connectivity: Clusters showing increased coupling during oral breathing compared to nasal breathing. **(a)** Axial slices showing significant clusters where connectivity with the brainstem seed was greater during oral breathing than nasal breathing. Results were thresholded at the voxel level (p < 0.01) and corrected at the cluster level (p-FDR < 0.05). Cold-colored overlays represent voxels with negative t-values (t(19)), where more negative values reflect stronger connectivity during oral breathing. Z-coordinates are labeled in MNI space for anatomical reference. **(b)** Glass brain views present maximum intensity projections of significant clusters from three perspectives: right lateral (top), superior (middle), and anterior (bottom). The color scale (range: −2.86 to −7.20) represents the magnitude of t-value, highlighting the spatial extent and bilateral distribution, primarily in limbic regions, of enhanced connectivity during oral breathing. All plots were generated using the CONN toolbox.

On the other hand, the first brainstem SBC cluster (4,586 voxels), centered in the right temporal region (+28, −20, −24), predominantly involved right-lateralized limbic and subcortical structures, including the hippocampus, parahippocampal cortex, amygdala, putamen, and insular cortex. The second brainstem SBC cluster (3,409 voxels) was localized to the left temporal region (−18, + 4, −24) and largely overlapped with the left-hemispheric counterparts of the regions identified in the first cluster. Fig 4a shows the spatial distribution of the significant clusters from the brainstem SBC analysis on axial slices. Fig 4b illustrates the maximum intensity projection statistics from the right lateral, superior, and anterior views, with emphasis on the voxel’s distribution of the affected limbic and subcortical regions. A summary of regions for which this clusters covers more than 10% of their volume is provided in Table 6, and the full composition details are listed in S3 and S4 Tables.

**Table 6:** Summary of significant clusters composition from the seed-based connectivity analysis of the brainstem (covering ≥ 10% of the regions).

Cluster 1				Cluster 2			
#	Region	Voxel Count	% Region Covered	#	Region	Voxel Count	% Region Covered
**1**	Hippocampus r	370	53	**1**	Hippocampus l	412	54
**2**	aPaHC r	367	56	**2**	Putamen l	327	38
**3**	Amygdala r	259	76	**3**	aPaHC l	266	46
**4**	Putamen r	252	31	**4**	IC l	232	17
**5**	IC r	226	17	**5**	Amygdala l	205	63
**6**	pTFusC r	189	26	**6**	pITG l	190	19
**7**	Caudate l	170	32	**7**	pTFusC l	175	20
**8**	aTFusC r	155	53	**8**	PP l	91	25
**9**	pPaHC r	91	29	**9**	pPaHC l	84	22
**10**	aITG r	33	10	**10**	aTFusC l	36	11

**Fig 5:**
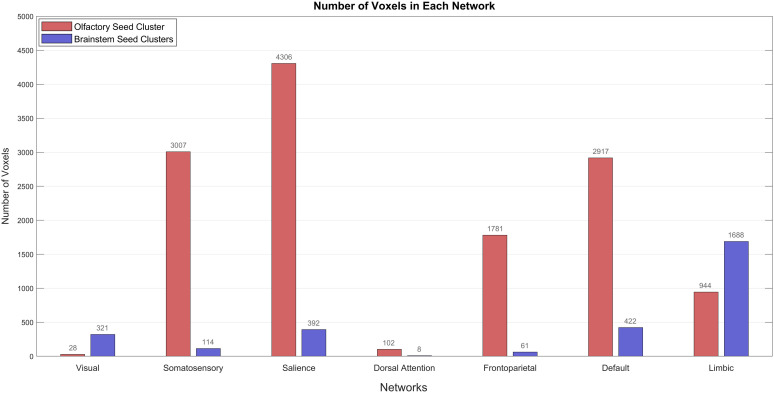
Distribution of voxels across functional brain networks in significant seed-based connectivity clusters. Bar plot showing the number of voxels within each large-scale brain network for significant clusters derived from seed-based connectivity analysis. Red bars represent the cluster associated with the olfactory seed, which shows strong engagement in the salience, somatosensory, default mode, frontoparietal, and limbic networks. Blue bars correspond to two significant clusters connected to the brainstem seed, with peak voxel counts in the limbic network. The bar height reflects voxel count, and numeric labels indicate the exact values. The plot was generated using MATLAB (The MathWorks Inc., USA).

The distribution of voxel counts from SBC clusters across functional networks, based on the Yeo seven-network parcellation [70], is provided in Fig 5 and detailed in S5 Table. The olfactory-seed cluster (Fig 5a), indicating broad and extensive network engagement (13,085 voxels), demonstrated markedly greater spatial coverage than the brainstem-seed clusters (3,006 voxels, Fig 5b). Specifically, the olfactory cluster showed widespread integration across the salience (4,306 voxels), somatosensory (3,007), default mode (2,917), frontoparietal (1,781), and limbic (944) networks. In contrast, the brainstem clusters were more restricted in scope, with the majority of their engagement localized to the limbic network (1,688 voxels), alongside limited representation in the default (422), salience (392), and visual (321) networks.

### 3.4. BOLD–physiological variability correlation

The BOLD–physiological variability correlation analysis revealed no statistically significant differences in the correlations between BOLD signals and either HRV or RVT regressors across all ROIs when comparing the nasal and oral breathing conditions (*p*-FDR > 0.05). S1 Fig displays the corrected p-values: panel (a) for BOLD–HRV, and panel (b) for BOLD–RVT.

### 3.5. Respiration–BOLD phase-locking validation

Reliable phase-locking between the respiration signal and BOLD time series was observed across all subjects. During nasal breathing, phase-locking between the respiratory signal and olfactory BOLD activity was reliably significant (%sigbins = 32.47% ± 9.64%; range: 17.45% to 57.82%). During oral breathing, significant phase-locking was also found between the respiratory signal and brainstem BOLD activity (%sigbins = 32.04% ± 10.24%; range: 14.91% to 50.55%). Examples of the original PLV time series and the corresponding significance threshold (*p* = 0.05) for representative subjects in both conditions are presented in S2 Fig. The complete dataset of %sigbins values for each breathing condition is provided in S4 Data.

The control analysis showed no significant differences between PLV results obtained (1) using the cleanly filtered respiratory signals and (2) from an intentionally aliased version. A paired permutation test yielded p-values of *p* = 0.80 for the nasal breathing condition (olfactory region) and *p* = 0.99 for the oral breathing condition (brainstem).

## 4. Discussion

### 4.1. Respiration-entrained BOLD oscillations

The central premise of this study is that BOLD oscillations in the IMF band (0.1–0.2 Hz) are genuinely entrained by respiration. Our phase-locking analysis reveals breathing mode-dependent temporal coupling between respiration and brain oscillations in this frequency band. Specifically, the reliable and statistically significant phase-locking between the respiratory rhythm and the olfactory BOLD signal during nasal breathing is consistent with OSNs functioning as the sensory entry point of the respiratory olfactory reafference (ROR) pathway [24]. This also aligns with the hypothesis that mechanical stimulation of OSNs by nasal airflow may serve as a primary mechanism for synchronizing cortical networks [21]. Conversely, the significant phase-locking observed between the respiratory rhythm and the brainstem BOLD signal during oral breathing suggests the presence of an internal pacemaker mechanism [19]. This is in line with the concept of a respiratory corollary discharge (RCD), an efference copy of the respiratory command generated in the brainstem and propagated to other brain regions [24]. Together, these findings provide converging evidence that the observed connectivity patterns are temporally coupled to the respiratory rhythm, supporting the conceptual framework of the REBON.

### 4.2. REBON: A novel breathing-mode dependent network

This study reveals that the mode of breathing selectively modulates the brain’s functional network. Using frequency-specific (0.1–0.2 Hz) resting-state fMRI analyses, we found that nasal breathing preferentially engages the olfactory region as a functional modulator, while oral breathing shifts network centrality toward the brainstem. These regions orchestrate distinct large-scale connectivity patterns, suggesting the existence of a respiration-entrained brain oscillation network (REBON) whose structure depends on breathing mode. Specifically, we define the REBON framework based on three operational criteria grounded in recent theoretical and our empirical work: (1) frequency specificity to the 0.1–0.2 Hz band (centered around ~0.16 Hz), (2) alternating dominance of modulatory nodes depending on breathing mode (olfactory region vs. brainstem), and (3) presence of a stable core of limbic and interoceptive structures.

The selection of the IMF band (0.1–0.2 Hz) was a theory-driven choice, guided by accumulating evidence on frequency-specific brain–body communication [22,23]. Unlike traditional fMRI studies focused on < 0.1 Hz, this band (centered around ~0.16 Hz) aligns with the binary hierarchy model [22], positioned between the slow oscillations (~0.08 Hz) and half the respiratory rhythm (~0.32 Hz). Empirical confirmation of respiration entrainment within this preferred frequency band represents a core property of the REBON framework.

The olfactory region and brainstem serve as principal modulators whose activity alternates in prominence depending on whether breathing occurs nasally or orally. Under nasal breathing, airflow continuously activates OSNs, stimulating the olfactory bulb and synchronizing oscillatory activity in the olfactory region, which in turn serve as a driver of brain functional connectivity [3,8,12]. As demonstrated in our results, this stimulation promotes coupling between the olfactory and higher-order regions, including the anterior cingulate cortex, frontal pole, opercular cortex, orbitofrontal cortex, inferior frontal gyrus, and paracingulate gyrus, which are functionally associated with cognitive control, emotion regulation, and interoceptive awareness [9,12,21,71,72].

In contrast, oral breathing bypasses the OSNs, leading to disengagement of the olfactory bulb’s rhythmic input [3,6]. In this state, a shift in whole-brain functional connectivity toward the brainstem, as the central respiratory pattern generator, becomes apparent [18]. Our findings show that oral breathing significantly increases connectivity between the brainstem and the basal ganglia, limbic, and thalamic regions, including the amygdala, hippocampus, insula, parahippocampal cortex, putamen, and thalamus, are well known for their roles in emotion regulation, autonomic integration, and survival-related behaviors [73,74]. One possible interpretation of the observed enhancement in brainstem-centered connectivity, in the absence of rhythmic olfactory input, is a reorganization toward a network architecture anchored in evolutionarily older systems, supporting physiological homeostasis and survival-oriented processing [18,75].

While the overall spatial topology of REBON is flexible, it is characterized by a core set of shared regions functionally connected to the respiration-entrained source node, notably the hippocampus, amygdala, and insula. These regions likely constitute the backbone of REBON, supporting fundamental aspects of respiration–brain interaction that persist regardless of breathing mode. These shared regions are deeply involved in interoception, autonomic control, and the integration of bodily rhythms with emotion and memory. For instance, the insula is the primary interoceptive cortex that monitors internal bodily states, such as respiratory sensations, emotion, and attention [76,77].

### 4.3. REBON and canonical resting-state networks

REBON is hypothesized to reflect neural entrainment to rhythmic respiration and is functionally distinct from canonical resting-state networks due to its sensitivity to breathing mode, particularly its mechanosensory origin via olfactory input. Nonetheless, seed-based connectivity analysis revealed and quantified areas of overlap between REBON and established resting-state networks (Fig 5). During nasal breathing, REBON exhibited widespread engagement of the salience, somatosensory, default, frontoparietal, and limbic networks, predominantly implicated in cognitive and executive functions [9,21,78]. In contrast, REBON during oral breathing was primarily confined to the limbic network, with more limited involvement of the default mode, salience, and visual networks. Notably, the limbic network emerged as the core shared component across both breathing conditions, though it was more prominently engaged during oral breathing. These results are strongly supported by recent findings in mice, which provide causal evidence that the ROR and RCD pathways independently contribute to the modulation of memory-related circuits in the hippocampus within the limbic system [24].

### 4.4. Applications of the REBON Framework

Given that our findings are inherently correlational, the proposed potential applications are hypothesis-generating and grounded in prior related studies. Nonetheless, they offer intriguing avenues for translational research. The REBON framework highlights the possibility that nasal respiration-entrained brain oscillations may serve as an effective entry point for the therapeutic modulation of large-scale brain networks. For instance, interventions such as slow nasal breathing (meditation practices), OSNs stimulation via rhythmic air-puffs, or even non-invasive stimulation of the nasal cavity may influence fronto-limbic circuitry involved in emotion regulation and cognitive control [15,16,79–81]. This suggests a potential role for REBON-targeted protocols in managing disorders characterized by dysregulated interoception or impaired network dynamics, such as anxiety, depression, insomnia, and neurodegenerative diseases. Future studies should integrate these interventions with neuroimaging and behavioral assessments to test whether enhancing the stability or flexibility of REBON could promote mental health and cognitive resilience across clinical and non-clinical populations.

### 4.5. Potential physiological and autonomic confounds

A critical methodological consideration in this study was how to handle physiological signals. While standard fMRI preprocessing pipelines often include the regression of physiological parameters such as HRV and RVT to remove potential sources of noise, we deliberately chose not to apply such regression. This decision was grounded in the primary goal of our study and is supported by a growing body of literature suggesting that respiration is not merely a physiological confound but a fundamental rhythm that actively entrains neural activity across widespread brain regions [13,21,54]. Given that HRV and RVT are closely linked to respiratory dynamics, regressing them out could potentially remove the neural signals related to respiration–brain coupling that we aim to investigate.

The BOLD–physiological correlation analysis was performed to assess whether unmodeled traditional physiological confounds might account for the observed differences between nasal and oral breathing. Our findings revealed no significant differences in BOLD–physiological correlations across the 133 ROIs between the nasal and oral breathing conditions. These results suggest that the observed connectivity differences are unlikely to be driven by residual physiological variability and are more likely attributable to genuine respiration-entrained neural dynamics.

The autonomic state, regulated by the autonomic nervous system (ANS), can also be a confounding factor. To address this, we employed a multi-level approach to assess autonomic variability across conditions. First, average RF and HR were compared between nasal and oral breathing using a paired permutation test. Neither measure showed a statistically significant difference, suggesting similar global physiological states. However, these broad metrics alone may not capture more subtle autonomic dynamics. Additionally, BOLD–physiological variability analysis revealed no significant differences in BOLD–HRV and BOLD–RVT correlations between conditions.

To more directly assess autonomic modulation, we computed the RMSSD, a HRV index that reflects parasympathetic activity and is widely used as a sensitive marker of vagal tone [34]. RMSSD values did not significantly differ between breathing modes, further suggesting comparable autonomic tone across conditions.

Notably, the observed RMSSD values were lower than those typically reported in previous studies [34,82]. These low RMSSD may reflect reduced parasympathetic tone and heightened sympathetic dominance, consistent with the scanner environment being an anxiety-inducing context, especially for individuals unfamiliar with MRI procedures [20,83]. This observation reinforces that while overall autonomic activity was low, it was similar between conditions.

Nonetheless, the potential influence of autonomic state cannot be fully excluded, particularly in the oral breathing condition, which may have induced respiratory discomfort, stress-related autonomic activation, and heightened arousal. Consequently, an alternative interpretation of the prominent brainstem activity observed during oral breathing is that it may reflect these unmodeled physiological and psychological factors. Future studies would benefit from in-scanner assessments (e.g., questionnaires) of discomfort, stress, and anxiety to better characterize and model potential autonomic confounds across conditions.

### 4.6. Addressing aliasing artifacts

An important general consideration in interpreting fMRI findings, and specifically the phase-locking between respiration and BOLD signals in our study, is the potential confounding influence of aliasing. Given the relatively low sampling rate of fMRI (TR = 2 s), higher-frequency physiological signals, particularly cardiac and respiratory rhythms, can be folded into lower frequency bands. This phenomenon raises the possibility that observed coupling in the 0.1–0.2 Hz range may reflect aliased components of faster physiological signals rather than genuine respiration-entrained neural oscillations. Although our frequency of interest was a narrow intermediate band, which minimizes the influence of broadband or high-frequency contamination, the potential for aliasing effects should still be addressed.

The results of our phase-locking control analysis showed no significant differences between the PLV values derived from the cleanly filtered respiratory signal and those from the intentionally aliased version. This indicates that the observed respiration–BOLD phase-locking effects were not artifacts of aliased signal content. Moreover, given the significant phase-locking between BOLD signals and the respiratory rhythm within the 0.1–0.2 Hz band, the possibility that cardiac aliasing contributed to the observed effects in this frequency range can be reasonably ruled out. This reasoning aligns with prior similar work, which used concordance with respiratory patterns to exclude aliasing effects from BOLD signals around ~0.16 Hz [23].

### 4.7. Limitations and future perspectives

While this study provides important insights, several limitations should be acknowledged. The primary limitation is the small, demographically homogeneous sample, which limits the generalizability of our findings to broader populations. Future research is therefore needed to validate the REBON framework in larger, more diverse populations, including females, individuals across a wider age range, and various clinical groups.

A further methodological consideration concerns our phase-locking analysis. Although the approach is robust, it was constrained by the temporal resolution of the fMRI data (TR = 2 s). This relatively low sampling rate limits the precision of instantaneous phase estimates of BOLD oscillations. Nevertheless, while the current findings strongly support respiration entrainment, future studies could benefit from replicating the analysis using data acquired at higher temporal resolution.

Furthermore, the results of this study relied solely on resting-state fMRI data, without accompanying behavioral, cognitive, or emotional task-based correlation analyses. Therefore, our interpretations regarding the functional roles of the observed networks in cognitive and emotional processes are preliminary, shaped by existing literature and the established roles of the identified brain networks. To verify these hypotheses, future research should employ task-based experimental designs and integrate behavioral measures specifically aimed at assessing the cognitive and emotional relevance of the observed network configurations.

This study also lacked direct measurements of transient state changes such as fatigue and arousal. While design considerations, including pre-run adaptation periods and inter-run rest breaks, were implemented to mitigate these effects, their influence cannot be entirely excluded. Similarly, fluctuations in blood gas exchange were not assessed. However, a recent review [21] suggests that differences in cognitive functions and brain activity between nasal and oral breathing are unlikely to stem from variations in gas exchange functions, which are expected to be similar between conditions. Future studies should incorporate direct measurements of these variables, along with self-report measures, to better account for these potential confounding factors and to further characterize the interaction between breathing mode and brain–body dynamics.

## 5. Conclusion

This study introduced REBON, a dynamic frequency-specific framework that reveals how the mode of breathing, nasal versus oral, modulates large-scale functional brain organization. Our findings showed that nasal breathing activates the olfactory region as a respiration-entrained node, engaging widespread connectivity across cognitive and emotional networks, while oral breathing shifts functional dominance toward the brainstem, reflecting a pattern more aligned with internal autonomic regulation. Despite these distinct configurations, both breathing modes engage a common limbic and interoceptive scaffold involving the hippocampus, amygdala, and insula, suggesting a stable backbone for respiration-brain interaction. Importantly, our findings, when considered alongside prior evidence, suggest the therapeutic potential of OSNs stimulation. Targeted activation via nasal breathing, rhythmic nasal air-puffs, or stimulation of the nasal cavity could provide a non-invasive alternative to deep brain stimulation, with the potential to support cognitive function, emotion regulation, and network resilience in both clinical and wellness contexts. The REBON framework may offer a valuable foundation for developing breath-based neuromodulation and understanding of the embodied dynamics of brain function.

## Supporting information

S1 Table List of 133 ROIs: abbreviations and full names. (DOCX)

S2 Table Full composition details of the significant cluster in the seed-based connectivity analysis of the olfactory. (DOCX)

S3 Table Full composition details of the first significant cluster in the seed-based connectivity analysis of the brainstem. (DOCX)

S4 Table Full composition details of the second significant cluster in the seed-based connectivity analysis of the brainstem. (DOCX)

S5 Table Number of voxels per network in the seed-based connectivity results: brainstem clusters, their combined total, and the olfactory cluster. (DOCX)

S1 Fig BOLD–physiological variability analysis across ROIs between breathing conditions. (a) ROI-wise analysis of the correlation between the BOLD signal and heart rate variability (HRV). (b) ROI-wise analysis of the correlation between the BOLD signal and respiratory volume per time (RVT). The horizontal axis shows ROI indices (1–133), and the vertical axis shows FDR-corrected p-values. Each blue dot represents the FDR-corrected p-value from a paired t-test for a single ROI. The red line indicates the significance threshold (*p*-FDR = 0.05). No ROIs showed a significant difference in BOLD–HRV or BOLD–RVT coupling between the two conditions. All plots were generated using MATLAB (The MathWorks Inc., USA).
(TIF)

S2 Fig Examples of phase locking between respiration and BOLD signals in representative subjects. (a) Phase locking between the nasal respiration signal and olfactory BOLD in Subject 20, with a significant proportion of time bins (%sigbins) equal to 47.27%. (b) Phase locking between the oral respiration signal and brainstem BOLD in Subject 1, with %sigbins equal to 49.09%. The black line represents the observed PLV time series; the gray line indicates the significance threshold (*p* = 0.05) derived from the null distribution generated using 1,000 surrogate datasets; and significant observed PLV are highlighted in magenta. All plots were generated using MATLAB (The MathWorks Inc., USA).
(TIF)

S1 Data Fisher Z-transformed ROI-to-ROI connectivity matrices for the nasal breathing condition. (XLSX)

S2 Data Fisher Z-transformed ROI-to-ROI connectivity matrices for the oral breathing condition. (XLSX)

S3 Data Physiological metrics: heart rate (HR), respiration frequency (RF), and root mean square of successive differences (RMSSD) for each breathing mode. (XLSX)

S4 Data Percentage of significant bins (%sigbins) for each breathing mode. (XLSX)
