# Supplementary material for: Breathing mode selectively modulates brain-wide functional connectivity
Source: PLoS One. 2025 Nov 14;20(11):e0334165. doi: 10.1371/journal.pone.0334165 (PMC12617844; doi:10.1371/journal.pone.0334165)
Supplement: S1 Table — (DOCX) [file pone.0334165.s001.docx]

**S1 Table. List of 133 ROIs: abbreviations and full names (continued).**

| **#** | **ROI Abbreviation** | **ROI Full Name** |
| --- | --- | --- |
| **1** | FP r | Frontal Pole Right |
| **2** | FP l | Frontal Pole Left |
| **3** | IC r | Insular Cortex Right |
| **4** | IC l | Insular Cortex Left |
| **5** | SFG r | Superior Frontal Gyrus Right |
| **6** | SFG l | Superior Frontal Gyrus Left |
| **7** | MidFG r | Middle Frontal Gyrus Right |
| **8** | MidFG l | Middle Frontal Gyrus Left |
| **9** | IFG tri r | Inferior Frontal Gyrus, pars triangularis Right |
| **10** | IFG tri l | Inferior Frontal Gyrus, pars triangularis Left |
| **11** | IFG oper r | Inferior Frontal Gyrus, pars opercularis Right |
| **12** | IFG oper l | Inferior Frontal Gyrus, pars opercularis Left |
| **13** | PreCG r | Precentral Gyrus Right |
| **14** | PreCG l | Precentral Gyrus Left |
| **15** | TP r | Temporal Pole Right |
| **16** | TP l | Temporal Pole Left |
| **17** | aSTG r | Superior Temporal Gyrus, anterior division Right |
| **18** | aSTG l | Superior Temporal Gyrus, anterior division Left |
| **19** | pSTG r | Superior Temporal Gyrus, posterior division Right |
| **20** | pSTG l | Superior Temporal Gyrus, posterior division Left |
| **21** | aMTG r | Middle Temporal Gyrus, anterior division Right |
| **22** | aMTG l | Middle Temporal Gyrus, anterior division Left |
| **23** | pMTG r | Middle Temporal Gyrus, posterior division Right |
| **24** | pMTG l | Middle Temporal Gyrus, posterior division Left |
| **25** | toMTG r | Middle Temporal Gyrus, temporooccipital part Right |
| **26** | toMTG l | Middle Temporal Gyrus, temporooccipital part Left |
| **27** | aITG r | Inferior Temporal Gyrus, anterior division Right |
| **28** | aITG l | Inferior Temporal Gyrus, anterior division Left |
| **29** | pITG r | Inferior Temporal Gyrus, posterior division Right |
| **30** | pITG l | Inferior Temporal Gyrus, posterior division Left |
| **31** | toITG r | Inferior Temporal Gyrus, temporooccipital part Right |
| **32** | toITG l | Inferior Temporal Gyrus, temporooccipital part Left |
| **33** | PostCG r | Postcentral Gyrus Right |
| **34** | PostCG l | Postcentral Gyrus Left |
| **35** | SPL r | Superior Parietal Lobule Right |
| **36** | SPL l | Superior Parietal Lobule Left |
| **37** | aSMG r | Supramarginal Gyrus, anterior division Right |
| **38** | aSMG l | Supramarginal Gyrus, anterior division Left |
| **39** | pSMG r | Supramarginal Gyrus, posterior division Right |
| **40** | pSMG l | Supramarginal Gyrus, posterior division Left |
| **41** | AG r | Angular Gyrus Right |
| **42** | AG l | Angular Gyrus Left |
| **43** | sLOC r | Lateral Occipital Cortex, superior division Right |
| **44** | sLOC l | Lateral Occipital Cortex, superior division Left |

**S1 Table. List of 133 ROIs: abbreviations and full names (continued).**

| **#** | **ROI Abbreviation** | **ROI Full Name** |
| --- | --- | --- |
| **45** | iLOC r | Lateral Occipital Cortex, inferior division Right |
| **46** | iLOC l | Lateral Occipital Cortex, inferior division Left |
| **47** | ICC r | Intracalcarine Cortex Right |
| **48** | ICC l | Intracalcarine Cortex Left |
| **49** | MedFC | Frontal Medial Cortex |
| **50** | SMA r | Juxtapositional Lobule Cortex - formerly Supplementary Motor Cortex - Right |
| **51** | SMA l | Juxtapositional Lobule Cortex - formerly Supplementary Motor Cortex - Left |
| **52** | SubCalC | Subcallosal Cortex |
| **53** | PaCiG r | Paracingulate Gyrus Right |
| **54** | PaCiG l | Paracingulate Gyrus Left |
| **55** | AC | Cingulate Gyrus, anterior division |
| **56** | PC | Cingulate Gyrus, posterior division |
| **57** | Precuneous | Precuneous Cortex |
| **58** | Cuneal r | Cuneal Cortex Right |
| **59** | Cuneal l | Cuneal Cortex Left |
| **60** | FOrb r | Frontal Orbital Cortex Right |
| **61** | FOrb l | Frontal Orbital Cortex Left |
| **62** | aPaHC r | Parahippocampal Gyrus, anterior division Right |
| **63** | aPaHC l | Parahippocampal Gyrus, anterior division Left |
| **64** | pPaHC r | Parahippocampal Gyrus, posterior division Right |
| **65** | pPaHC l | Parahippocampal Gyrus, posterior division Left |
| **66** | LG r | Lingual Gyrus Right |
| **67** | LG l | Lingual Gyrus Left |
| **68** | aTFusC r | Temporal Fusiform Cortex, anterior division Right |
| **69** | aTFusC l | Temporal Fusiform Cortex, anterior division Left |
| **70** | pTFusC r | Temporal Fusiform Cortex, posterior division Right |
| **71** | pTFusC l | Temporal Fusiform Cortex, posterior division Left |
| **72** | TOFusC r | Temporal Occipital Fusiform Cortex Right |
| **73** | TOFusC l | Temporal Occipital Fusiform Cortex Left |
| **74** | OFusG r | Occipital Fusiform Gyrus Right |
| **75** | OFusG l | Occipital Fusiform Gyrus Left |
| **76** | FO r | Frontal Operculum Cortex Right |
| **77** | FO l | Frontal Operculum Cortex Left |
| **78** | CO r | Central Opercular Cortex Right |
| **79** | CO l | Central Opercular Cortex Left |
| **80** | PO r | Parietal Operculum Cortex Right |
| **81** | PO l | Parietal Operculum Cortex Left |
| **82** | PP r | Planum Polare Right |
| **83** | PP l | Planum Polare Left |
| **84** | HG r | Heschl's Gyrus Right |
| **85** | HG l | Heschl's Gyrus Left |
| **86** | PT r | Planum Temporale Right |
| **87** | PT l | Planum Temporale Left |
| **88** | SCC r | Supracalcarine Cortex Right |

**S1 Table. List of 133 ROIs: abbreviations and full names.**

| **#** | **ROI Abbreviation** | **ROI Full Name** |
| --- | --- | --- |
| **89** | SCC l | Supracalcarine Cortex Left |
| **90** | OP r | Occipital Pole Right |
| **91** | OP l | Occipital Pole Left |
| **92** | Thalamus r | Thalamus Right |
| **93** | Thalamus l | Thalamus Left |
| **94** | Caudate r | Caudate Right |
| **95** | Caudate l | Caudate Left |
| **96** | Putamen r | Putamen Right |
| **97** | Putamen l | Putamen Left |
| **98** | Pallidum r | Pallidum Right |
| **99** | Pallidum | Pallidum Left |
| **100** | Hippocampus r | Hippocampus Right |
| **101** | Hippocampus l | Hippocampus Left |
| **102** | Amygdala r | Amygdala Right |
| **103** | Amygdala l | Amygdala Left |
| **104** | Accumbens | Accumbens Right |
| **105** | Accumbens l | Accumbens Left |
| **106** | Brainstem | Brainstem |
| **107** | Cereb1 | Cerebellum Crus1 Left |
| **108** | Cereb1 | Cerebellum Crus1 Right |
| **109** | Cereb2 l | Cerebellum Crus2 Left |
| **110** | Cereb2 r | Cerebellum Crus2 Right |
| **111** | Cereb3 l | Cerebellum 3 Left |
| **112** | Cereb3 r | Cerebellum 3 Right |
| **113** | Cereb45 l | Cerebellum 4/5 Left |
| **114** | Cereb45 r | Cerebellum 4/5 Right |
| **115** | Cereb6 l | Cerebellum 6 Left |
| **116** | Cereb6 r | Cerebellum 6 Right |
| **117** | Cereb7 l | Cerebellum 7b Left |
| **118** | Cereb7 r | Cerebellum 7b Right |
| **119** | Cereb8 l | Cerebellum 8 Left |
| **120** | Cereb8 r | Cerebellum 8 Right |
| **121** | Cereb9 l | Cerebellum 9 Left |
| **122** | Cereb9 r | Cerebellum 9 Right |
| **123** | Cereb10 l | Cerebellum 10 Left |
| **124** | Cereb10 r | Cerebellum 10 Right |
| **125** | Ver12 | Vermis 1/2 |
| **126** | Ver3 | Vermis 3 |
| **127** | Ver45 | Vermis 4/5 |
| **128** | Ver6 | Vermis 6 |
| **129** | Ver7 | Vermis 7 |
| **130** | Ver8 | Vermis 8 |
| **131** | Ver9 | Vermis 9 |
| **132** | Ver10 | Vermis 10 |
| **133** | Olfactory | Olfactory Cortex (Left and Right) |
