# Supplementary material for: Breathing mode selectively modulates brain-wide functional connectivity
Source: PLoS One. 2025 Nov 14;20(11):e0334165. doi: 10.1371/journal.pone.0334165 (PMC12617844; doi:10.1371/journal.pone.0334165)
Supplement: S2 Table — (DOCX) [file pone.0334165.s002.docx]

**S2 Table. Full composition details of the significant cluster in the seed-based connectivity analysis of the olfactory (continued).**

| **#** | **Region** | **Voxel Count** | **Percent Total** | **% Region Covered** | **Center** | | |
| --- | --- | --- | --- | --- | --- | --- | --- |
|  |  |  |  |  | **X** | **Y** | **Z** |
| **1** | AC | 1052 | 5 | 41 | +2 | 18 | 26 |
| **2** | IC l | 905 | 4 | 68 | -36 | +2 | 0 |
| **3** | FP r | 838 | 4 | 10 | +34 | +48 | +6 |
| **4** | IC r | 766 | 3 | 57 | +36 | +6 | 0 |
| **5** | Brainstem | 709 | 3 | 17 | 0 | -30 | -38 |
| **6** | Putamen l | 650 | 3 | 75 | -26 | +2 | +2 |
| **7** | CO r | 552 | 2 | 63 | +50 | -6 | +12 |
| **8** | CO l | 550 | 2 | 56 | -46 | -4 | +10 |
| **9** | PaCiG l | 541 | 2 | 41 | -8 | +36 | +24 |
| **10** | Putamen r | 513 | 2 | 64 | +26 | +4 | 0 |
| **11** | TP r | 499 | 2 | 21 | +40 | +12 | -24 |
| **12** | FP l | 446 | 2 | 6 | -28 | +48 | +10 |
| **13** | PreCG r | 429 | 2 | 10 | +46 | -4 | +44 |
| **14** | PaCiG r | 405 | 2 | 30 | +10 | +36 | +24 |
| **15** | FOrb l | 393 | 2 | 23 | -36 | +26 | -12 |
| **16** | FOrb r | 337 | 1 | 23 | +36 | +24 | -10 |
| **17** | PO r | 337 | 1 | 63 | +50 | -26 | +20 |
| **18** | SFG r | 330 | 1 | 12 | +16 | +32 | +46 |
| **19** | FO r | 281 | 1 | 90 | +42 | +18 | +6 |
| **20** | MidFG r | 275 | 1 | 10 | +32 | +24 | +42 |
| **21** | TP l | 272 | 1 | 11 | -46 | +8 | -16 |
| **22** | SMA r | 236 | 1 | 33 | +8 | -6 | +52 |
| **23** | PostCG r | 230 | 1 | 7 | +48 | -16 | +38 |
| **24** | Hippocampus r | 228 | 1 | 33 | +26 | -14 | -20 |
| **25** | IFG tri r | 221 | 1 | 40 | +52 | +26 | +2 |
| **26** | Amygdala l | 217 | 1 | 66 | -26 | -4 | -18 |
| **27** | PT r | 209 | 1 | 48 | +56 | -22 | 12 |
| **28** | Hippocampus l | 203 | 1 | 27 | -24 | -14 | -20 |
| **29** | IFG oper r | 195 | 1 | 28 | +52 | +16 | +8 |
| **30** | FO l | 190 | 1 | 54 | -40 | +16 | +4 |
| **31** | PP l | 190 | 1 | 53 | -46 | -4 | -8 |
| **32** | Amygdala r | 187 | 1 | 55 | +26 | -2 | -20 |
| **33** | aPaHC l | 184 | 1 | 32 | -22 | -4 | -32 |
| **34** | aPaHC r | 175 | 1 | 27 | 24 | -6 | -34 |
| **35** | PO l | 162 | 1 | 29 | -48 | -30 | +22 |
| **36** | SFG l | 152 | 1 | 5 | -6 | +44 | +34 |
| **37** | HG l | 152 | 1 | 49 | -46 | -20 | +8 |
| **38** | SMA L | 148 | 1 | 23 | -4 | -6 | +62 |
| **39** | PP r | 139 | 1 | 37 | +48 | -4 | -8 |
| **40** | Caudate l | 129 | 1 | 24 | -16 | +14 | +8 |

**S2 Table. Full composition details of the significant cluster in the seed-based connectivity analysis of the olfactory.**

| **#** | **Region** | **Voxel Count** | **Percent Total** | **% Region Covered** | **Center** | | |
| --- | --- | --- | --- | --- | --- | --- | --- |
|  |  |  |  |  | **X** | **Y** | **Z** |
| **41** | PreCG l | 119 | 1 | 3 | -14 | -14 | +68 |
| **42** | IFG tri l | 111 | 0 | 17 | -50 | +26 | +12 |
| **43** | aSTG r | 83 | 0 | 30 | +54 | 0 | -12 |
| **44** | aTFusC r | 83 | 0 | 28 | +28 | -2 | -40 |
| **45** | Pallidum l | 75 | 0 | 25 | -20 | -4 | +2 |
| **46** | IFG oper l | 74 | 0 | 10 | -50 | +12 | +2 |
| **47** | PC | 66 | 0 | 3 | +10 | -24 | +40 |
| **48** | pMTG r | 63 | 0 | 5 | +52 | -12 | -16 |
| **49** | Thalamus r | 63 | 0 | 5 | +16 | -16 | +6 |
| **50** | HG r | 62 | 0 | 22 | +50 | -16 | +6 |
| **51** | Cereb8 r | 55 | 0 | 2 | 24 | -46 | -58 |
| **52** | Thalamus l | 52 | 0 | 4 | -10 | -10 | +2 |
| **53** | Caudate r | 49 | 0 | 9 | +16 | +14 | +10 |
| **54** | aSTG l | 39 | 0 | 14 | -52 | -8 | -10 |
| **55** | aSMG r | 39 | 0 | 5 | +58 | -26 | +26 |
| **56** | PT l | 38 | 0 | 7 | -52 | -30 | +10 |
| **57** | pSMG r | 37 | 0 | 3 | 66 | -38 | +18 |
| **58** | Cereb9 r | 34 | 0 | 4 | +12 | -44 | -54 |
| **59** | MidFG l | 28 | 0 | 1 | -26 | +30 | +32 |
| **60** | pPaHC r | 24 | 0 | 8 | +22 | -32 | -14 |
| **61** | pSTG r | 23 | 0 | 6 | +54 | -18 | -4 |
| **62** | aMTG r | 22 | 0 | 5 | +56 | -4 | -18 |
| **63** | PostCG l | 19 | 0 | 1 | -62 | -8 | +16 |
| **64** | Pallidum r | 14 | 0 | 5 | +22 | -10 | +4 |
| **65** | aTFusC l | 9 | 0 | 3 | -28 | -4 | -38 |
| **66** | aMTG l | 8 | 0 | 2 | -54 | -8 | -14 |
| **67** | pPaHC l | 8 | 0 | 2 | -12 | -38 | -6 |
| **68** | Cereb45 l | 8 | 0 | 1 | -8 | -36 | -6 |
| **69** | pSTG l | 6 | 0 | 2 | -60 | -36 | +10 |
| **70** | Precuneous | 4 | 0 | 0 | +14 | -36 | +44 |
| **71** | Cereb3 l | 3 | 0 | 2 | -6 | -36 | -10 |
| **72** | Cereb9 l | 3 | 0 | 0 | -10 | -42 | -50 |
| **73** | pTFusC r | 2 | 0 | 0 | +30 | -12 | -38 |
| **74** | pMTG l | 1 | 0 | 0 | -52 | -10 | -16 |
| **75** | aSMG l | 1 | 0 | 0 | -58 | -32 | +26 |
| **76** | MedFC | 1 | 0 | 0 | +8 | +52 | -6 |
| **77** | pTFusC l | 1 | 0 | 0 | -36 | -16 | -24 |
| **78** | not-labeled | 6794 | 30 | 2 | +14 | +10 | +6 |
