# Supplementary material for: Breathing mode selectively modulates brain-wide functional connectivity
Source: PLoS One. 2025 Nov 14;20(11):e0334165. doi: 10.1371/journal.pone.0334165 (PMC12617844; doi:10.1371/journal.pone.0334165)
Supplement: S3 Table — (DOCX) [file pone.0334165.s003.docx]

**S3 Table. Full composition details of the first significant cluster in the seed-based connectivity analysis of the brainstem.**

| **#** | **Region** | **Voxel Count** | **Percent Total** | **% Region Covered** | **Center** | | |
| --- | --- | --- | --- | --- | --- | --- | --- |
|  |  |  |  |  | **X** | **Y** | **Z** |
| **1** | Hippocampus r | 370 | 8 | 53 | 28 | -16 | -20 |
| **2** | aPaHC r | 367 | 8 | 56 | 26 | -8 | -30 |
| **3** | Amygdala r | 259 | 6 | 76 | 24 | -4 | -18 |
| **4** | Putamen r | 252 | 5 | 31 | 28 | 2 | -4 |
| **5** | Brainstem | 241 | 5 | 6 | 4 | -26 | -26 |
| **6** | IC r | 226 | 5 | 17 | 36 | 6 | -2 |
| **7** | pTFusC r | 189 | 4 | 26 | 38 | -16 | -30 |
| **8** | Caudate l | 170 | 4 | 32 | -14 | 10 | 12 |
| **9** | aTFusC r | 155 | 3 | 53 | 32 | -4 | -40 |
| **10** | pPaHC r | 91 | 2 | 29 | 24 | -32 | -16 |
| **11** | FOrb r | 86 | 2 | 6 | 28 | 14 | -16 |
| **12** | TP r | 74 | 2 | 3 | 36 | 10 | -30 |
| **13** | pITG r | 60 | 1 | 6 | 46 | -18 | -30 |
| **14** | Caudate r | 43 | 1 | 8 | 12 | 10 | 8 |
| **15** | Thalamus r | 39 | 1 | 3 | 8 | -16 | 4 |
| **16** | aITG r | 33 | 1 | 10 | 44 | -6 | -40 |
| **17** | LG r | 27 | 1 | 2 | 22 | -40 | -10 |
| **18** | aSTG r | 23 | 1 | 8 | 52 | -4 | -14 |
| **19** | Cereb45 r | 22 | 0 | 4 | 16 | -38 | -18 |
| **20** | pMTG r | 19 | 0 | 1 | 50 | -14 | -14 |
| **21** | Thalamus l | 19 | 0 | 1 | -6 | -8 | 12 |
| **22** | Cereb3 r | 13 | 0 | 7 | 10 | -38 | -18 |
| **23** | aMTG r | 9 | 0 | 2 | 54 | -6 | -20 |
| **24** | CO r | 8 | 0 | 1 | 42 | -2 | 16 |
| **25** | PP r | 6 | 0 | 2 | 50 | 2 | -12 |
| **26** | PaCiG l | 5 | 0 | 0 | -12 | 22 | 32 |
| **27** | Putamen l | 3 | 0 | 0 | -20 | 6 | 6 |
| **28** | Pallidum r | 2 | 0 | 1 | 16 | 8 | -2 |
| **29** | pSTG r | 1 | 0 | 0 | 50 | -10 | -14 |
| **30** | PC | 1 | 0 | 0 | 20 | -42 | -2 |
| **31** | not-labeled | 1773 | 39 | 0 | 20 | -8 | -10 |
