# Supplementary material for: Breathing mode selectively modulates brain-wide functional connectivity
Source: PLoS One. 2025 Nov 14;20(11):e0334165. doi: 10.1371/journal.pone.0334165 (PMC12617844; doi:10.1371/journal.pone.0334165)
Supplement: S4 Table — (DOCX) [file pone.0334165.s004.docx]

**S4 Table. Full composition details of the second significant cluster in the seed-based connectivity analysis of the brainstem.**

| **#** | **Region** | **Voxel Count** | **Percent Total** | **% Region Covered** | **Center** | | |
| --- | --- | --- | --- | --- | --- | --- | --- |
|  |  |  |  |  | **X** | **Y** | **Z** |
| **1** | Hippocampus l | 412 | 12 | 54 | -26 | -20 | -16 |
| **2** | Putamen l | 327 | 10 | 38 | -28 | 0 | -2 |
| **3** | aPaHC l | 266 | 8 | 46 | -22 | -6 | -32 |
| **4** | IC l | 232 | 7 | 17 | -36 | 2 | 2 |
| **5** | Amygdala l | 205 | 6 | 63 | -26 | -4 | -20 |
| **6** | TP l | 199 | 6 | 8 | -36 | 8 | -26 |
| **7** | pITG l | 190 | 6 | 19 | -52 | -20 | -28 |
| **8** | pTFusC l | 175 | 5 | 20 | -38 | -20 | -28 |
| **9** | FOrb l | 115 | 3 | 7 | -26 | 12 | -22 |
| **10** | PP l | 91 | 3 | 25 | -46 | -6 | -12 |
| **11** | pPaHC l | 84 | 2 | 22 | -26 | -36 | -14 |
| **12** | LG l | 62 | 2 | 4 | -22 | -44 | -6 |
| **13** | aTFusC l | 36 | 1 | 11 | -30 | -8 | -38 |
| **14** | aSTG l | 26 | 1 | 9 | -50 | -6 | -14 |
| **15** | Pallidum l | 26 | 1 | 9 | -22 | -4 | -2 |
| **16** | TOFusC l | 17 | 0 | 3 | -30 | -46 | -10 |
| **17** | pMTG l | 11 | 0 | 1 | -58 | -16 | -26 |
| **18** | FO l | 8 | 0 | 2 | -36 | 18 | 4 |
| **19** | aMTG l | 3 | 0 | 1 | -48 | 0 | -28 |
| **20** | aITG l | 2 | 0 | 1 | -44 | -10 | -38 |
| **21** | PC | 2 | 0 | 0 | -16 | -44 | -2 |
| **22** | CO l | 1 | 0 | 0 | -42 | 0 | 8 |
| **23** | not-labeled | 919 | 27 | 0 | -30 | -6 | -16 |
