# Supplementary material for: Breathing mode selectively modulates brain-wide functional connectivity
Source: PLoS One. 2025 Nov 14;20(11):e0334165. doi: 10.1371/journal.pone.0334165 (PMC12617844; doi:10.1371/journal.pone.0334165)
Supplement: S5 Table — (DOCX) [file pone.0334165.s005.docx]

**S5 Table. Number of voxels per network in the seed-based connectivity results: brainstem clusters, their combined total, and the olfactory cluster.**

| **Network** | **Brainstem (Cluster 1)** | **Brainstem  (Cluster 2)** | **Brainstem  (total)** | **Olfactory** |
| --- | --- | --- | --- | --- |
| **Frontoparietal** | 46 | 15 | 61 | 1781 |
| **Default** | 143 | 279 | 422 | 2917 |
| **Limbic** | 912 | 776 | 1688 | 944 |
| **Visual** | 126 | 195 | 321 | 28 |
| **Somatosensory** | 83 | 31 | 114 | 3007 |
| **Salience** | 110 | 282 | 392 | 4306 |
| **Dorsal Attention** | 1 | 7 | 8 | 102 |
| **Total** | 1421 | 1585 | 3006 | 13085 |
