# Supplementary figures and images for: Breathing mode selectively modulates brain-wide functional connectivity
Source: PLoS One. 2025 Nov 14;20(11):e0334165. doi: 10.1371/journal.pone.0334165 (PMC12617844; doi:10.1371/journal.pone.0334165)

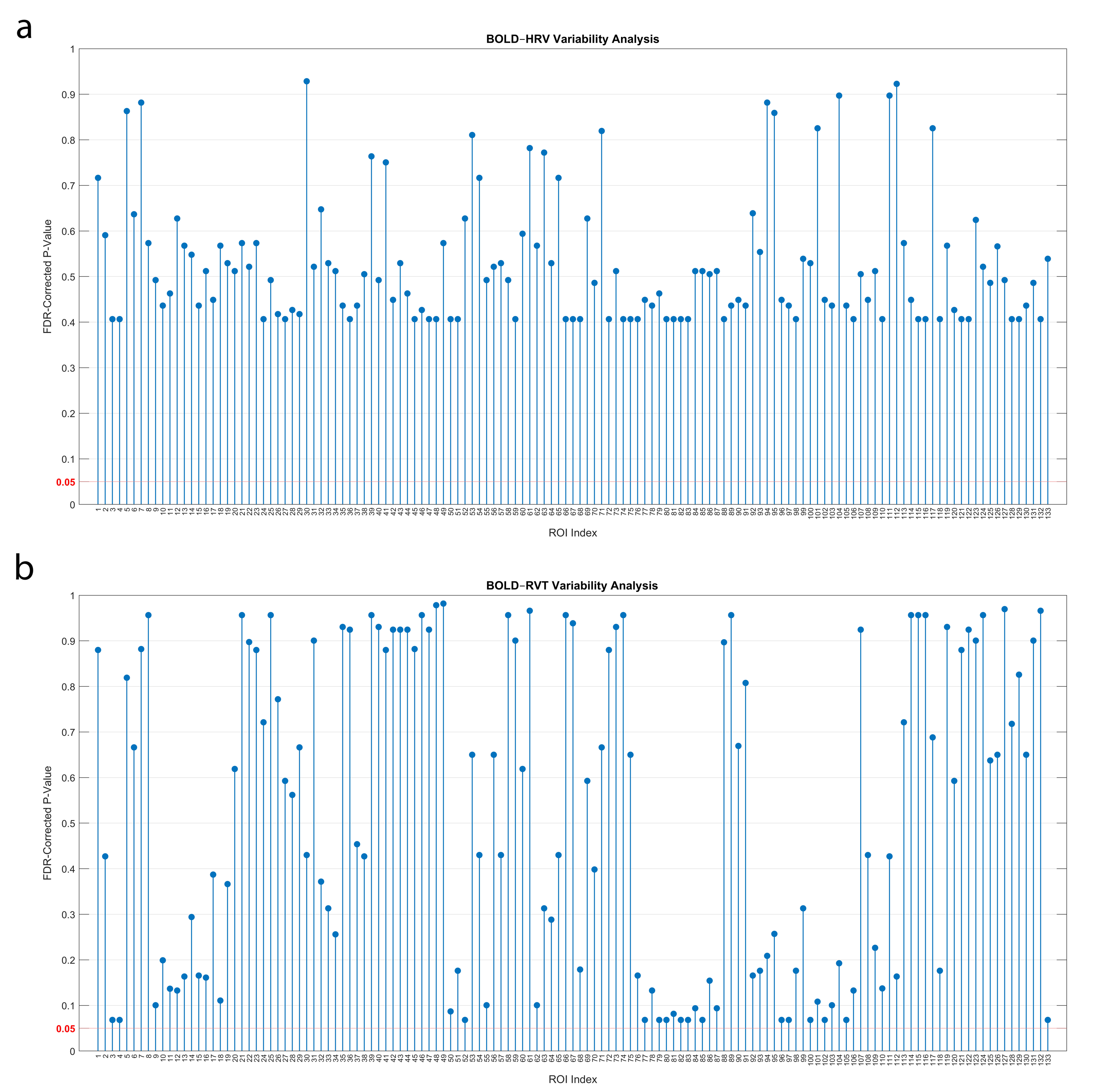

Supplement: S1 Fig — (a) ROI-wise analysis of the correlation between the BOLD signal and heart rate variability (HRV). (b) ROI-wise analysis of the correlation between the BOLD signal and respiratory volume per time (RVT). The horizontal axis shows ROI indices (1–133), and the vertical axis shows FDR-corrected p-values. Each blue dot represents the FDR-corrected p-value from a paired t-test for a single ROI. The red line indicates the significance threshold (p-FDR = 0.05). No ROIs showed a significant difference in BOLD–HRV or BOLD–RVT coupling between the two conditions. All plots were generated using MATLAB (The MathWorks Inc., USA). (TIF) [file pone.0334165.s006.tif]

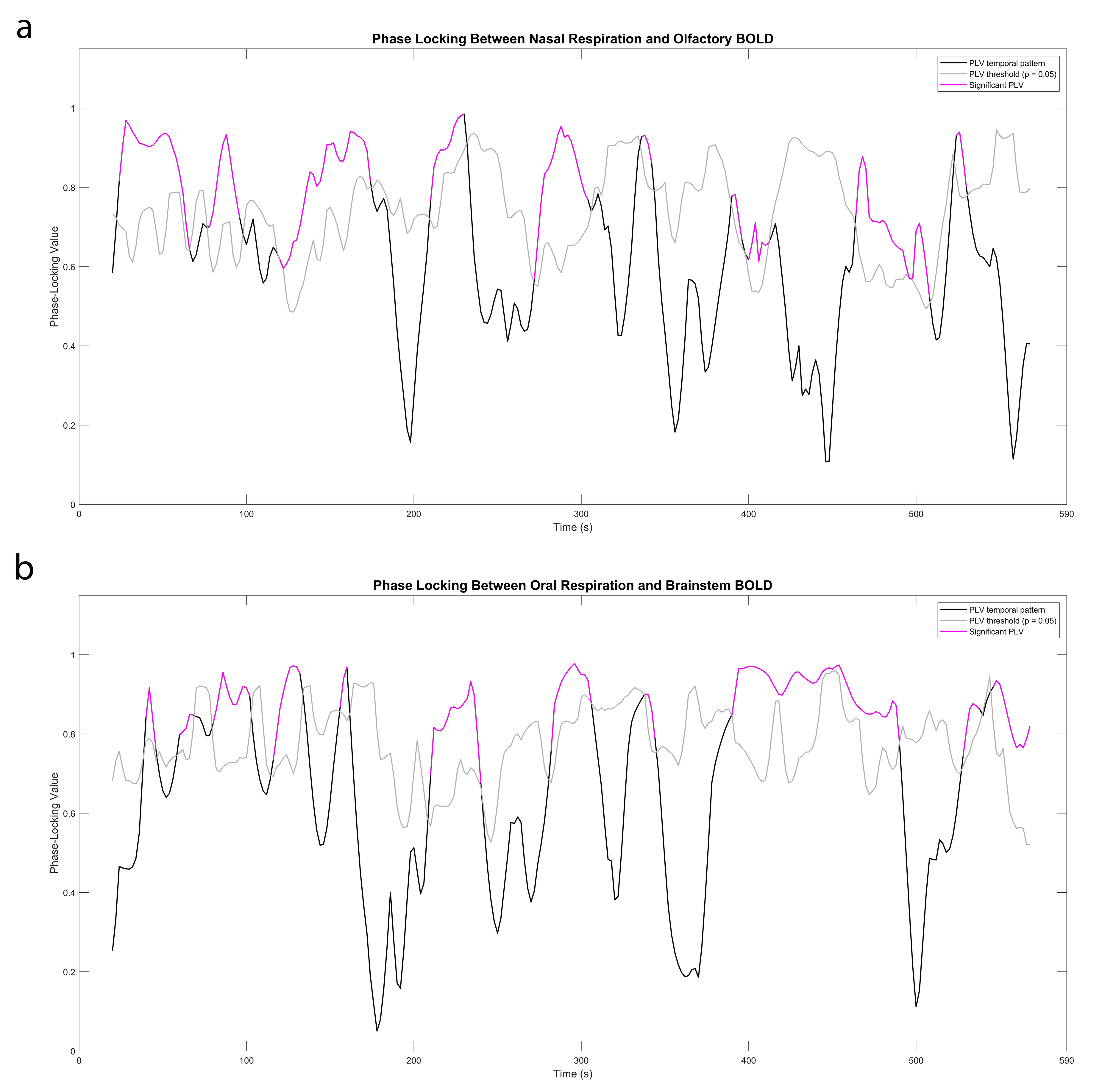

Supplement: S2 Fig — (a) Phase locking between the nasal respiration signal and olfactory BOLD in Subject 20, with a significant proportion of time bins (%sigbins) equal to 47.27%. (b) Phase locking between the oral respiration signal and brainstem BOLD in Subject 1, with %sigbins equal to 49.09%. The black line represents the observed PLV time series; the gray line indicates the significance threshold (p = 0.05) derived from the null distribution generated using 1,000 surrogate datasets; and significant observed PLV are highlighted in magenta. All plots were generated using MATLAB (The MathWorks Inc., USA). (TIF) [file pone.0334165.s007.tif]
